# Difficulties in Differentiating Coronaviruses from Subcellular Structures in Human Tissues by Electron Microscopy

**DOI:** 10.3201/eid2704.204337

**Published:** 2021-04

**Authors:** Hannah A. Bullock, Cynthia S. Goldsmith, Sherif R. Zaki, Roosecelis B. Martines, Sara E. Miller

**Affiliations:** Synergy America, Inc., Atlanta, Georgia, USA (H.A. Bullock);; Centers for Disease Control and Prevention, Atlanta (C.S. Goldsmith, S.R. Zaki, R.B. Martines);; Duke Medical Center, Durham, North Carolina, USA (S.E. Miller)

**Keywords:** COVID-19, SARS-CoV-2, severe acute respiratory syndrome coronavirus 2, viruses, respiratory infections, zoonoses, coronavirus disease, SARS, ultrastructure, coronavirus, electron microscopy

## Abstract

Efforts to combat the coronavirus disease (COVID-19) pandemic caused by severe acute respiratory syndrome coronavirus 2 (SARS-CoV-2) have placed a renewed focus on the use of transmission electron microscopy for identifying coronavirus in tissues. In attempts to attribute pathology of COVID-19 patients directly to tissue damage caused by SARS-CoV-2, investigators have inaccurately reported subcellular structures, including coated vesicles, multivesicular bodies, and vesiculating rough endoplasmic reticulum, as coronavirus particles. We describe morphologic features of coronavirus that distinguish it from subcellular structures, including particle size range (60–140 nm), intracellular particle location within membrane-bound vacuoles, and a nucleocapsid appearing in cross section as dense dots (6–12 nm) within the particles. In addition, although the characteristic spikes of coronaviruses may be visible on the virus surface, especially on extracellular particles, they are less evident in thin sections than in negative stain preparations.

The *Coronaviridae* family of viruses contains several human pathogens, including severe acute respiratory syndrome coronavirus 2 (SARS-CoV-2), the causative agent of the coronavirus disease (COVID-19) pandemic. Since early 2020, the unprecedented collective response to the COVID-19 pandemic from the scientific and medical community has led to numerous SARS-CoV-2–related publications and underscored the urgent need to demonstrate and verify the presence of coronavirus directly in tissues. Among these publications are reports describing the pathology of SARS-CoV-2 infection in patient specimens, which have been scrutinized intensely by electron microscopy (EM) for evidence of the virus. Consequently, several articles have erroneously described the identification of coronavirus particles by EM in the lung (*1*–*6*), kidney (*6*–*13*; B. Diao et al., unpub. data, https://doi.org/10.1101/2020.03.04.20031120), heart (*14**,**15*), brain (*16*), liver (*17*), intestine (*6*,*18*), skin (*19*), and placenta (*20*–*22*) (Table). However, most of the presumed virus or virus-like particles shown in all of these reports either represent normal subcellular organelles previously demonstrated in cells (*23*) or, otherwise, lack sufficient ultrastructure and morphologic features to be conclusively identified as coronavirus. Since early May 2020, letters to the editors of several journals have refuted these descriptions (*24*–*30*), yet the misidentification of coronavirus particles continues. It is essential for our collective understanding of COVID-19 clinical pathology and pathogenesis as well as the field of diagnostic EM that these misidentifications of SARS-CoV-2 particles be addressed.

**Table:** Structures misidentified as coronavirus particles by transmission electron microscopy in publications, March–November 2020*

Original reference	Tissue	Structures misidentified as coronavirus (Figure no., panel)	Correct identification	Response
Yao et al. (*1*)	Lung	Spiked vesicles in the cytoplasm (1, A–C)	CCVs	NA
Pesaresi et al. (*2*)	Lung	Vacuole containing vesicles (1, A, B)	MVB	NA
		Clusters of dark particles some associated with membranes (1, C, D)	RER and possibly ribosomes	
		Clusters of dark particles (2, A, B, E)	Unidentifiable structures	
Grimes et al. (*3*)	Lung	Vacuole containing vesicles (2, A)	MVB	NA
		Spiked vesicle in cytoplasm (2, B)	Possible CCV	
Ackermann et al. (*4*)	Lung	Dark circular structures (3, D)	Unidentifiable structure	Scholkmann et al. (*29*)
Borczuk et al. (*5*)	Lung	Clusters of dark particles associated with membranes (6, E)	Vesiculating RER	NA
		Spiked vesicle in cytoplasm (7, F)	CCV	
Bradley et al. (*6*)	Lung	Collections of vesicles (5, A, D)	Unidentifiable structures	Dittmayer et al. (*30*)
		Coated vesicles (5, B)	CCVs	
		Vacuole containing vesicles (5, C)	MVB	
	Intestine	Circular membranes in cytoplasm (5, E)	Unidentifiable structures	NA
		Extracellular spiked vesicles (5, F)	Unidentifiable structures	
	Kidney	Spiked vesicles within a membrane (5, G)	CCVs	NA
		Membrane bound vesicles (5, H)	Unidentifiable structures	
Su et al. (*7*)	Kidney	Spiked vesicles in cytoplasm (2, A–D)	CCVs	Calomeni et al. (*24*); Miller et al. (*27*); Roufosse et al. (*28*)
Kissling et al. (*8*)	Kidney	Vacuole containing vesicles (1, E, F)	MVB	Calomeni et al. (*24*); Miller et al. (*27*); Roufosse et al. (*28*)
Varga et al. (*9*)	Kidney	Circular membrane structures with surrounding black dots (1, A, B)	Vesiculating RER	Goldsmith et al. (*26*); Roufosse et al. (*28*)
Farkash et al. (*10*)	Kidney	Spiked vesicles in cytoplasm (3, A–C)	CCVs	Miller et al. (*25*); Roufosse et al. (*28*)
		Vacuole containing vesicles (3, D)	MVB	
B. Diao et al., unpub. data, https://doi.org/10.1101/2020.03.04.20031120	Kidney	Spiked vesicles in cytoplasm (3)	CCVs	Roufosse et al. (*28*)
Abbate et al. (*11*)	Kidney	Spiked vesicle in cytoplasm (1)	CCV	NA
Menter et al. (*12*)	Kidney	Vacuoles containing vesicles (4, A–C)	MVB,	NA
		Collection of membrane bound particles (4, D)	Unidentifiable structure	
Werion et al. (*13*)	Kidney	Circular vesicles with internal black dots (3, A–C)	Outside-in RER	NA
Tavazzi et al. (*14*)	Heart	Spiked vesicles in cytoplasm (2, A–F)	CCVs	Dittmayer et al. (*30*)
Dolhnikoff et al. (*15*)	Heart	Roughly circular black structures (3, A, D)	Unidentifiable structures	Dittmayer et al. (*30*)
		Clusters of dark particles, some associated with membranes (3, B, C)	RER and clusters of ribosomes	
Paniz-Mondolfi et al. (*16*)	Brain	Vacuole containing circular particles (3, A, B)	Unidentifiable structures	NA
		Vacuole containing vesicles (1, C, D)	MVB	
Wang et al. (*17*)	Liver	Circular structures with surrounding black dots (1, M; 2, J)	Vesiculating RER	NA
Qian et al. (*18*)	Intestine	Spiked vesicles in cytoplasm (3, A, B)	CCVs	NA
Colmenero et al. (*19*)	Skin	Spiked vesicle in cytoplasm (4, D)	CCV	NA
Hosier et al. (*20*)	Placenta	Spiked vesicles in cytoplasm (4, C–F)	CCVs	NA
		Spherical particles (4, G–I)	Unidentifiable structures	
Algarroba et al. (*21*)	Placenta	Spiked vesicles in cytoplasm (2–6)	CCVs	NA
Sisman et al. (*22*)	Placenta	Vacuole containing circular particles (1, C)	Unidentifiable structures	NA

*CCV, clathrin or coatomer coated vesicles; MVB, multivesicular body; NA, not applicable; RER, rough endoplasmic reticulum.

As of November 2020, only 2 articles and 1 letter to the editor had been published documenting clear EM evidence of SARS-CoV-2 directly in tissue samples (*30*–*32*), and another 2 articles showed rare viral particles (*33**,**34*). Here, we review published articles that used EM to search for SARS-CoV-2 in patient tissue samples. Our goal is to highlight the importance of coronavirus morphology and cellular localization in diagnosis and detection. In addition, we provide a side-by-side comparison of the subcellular structures that have been most frequently misinterpreted as SARS-CoV-2 along with actual viral particles that have been identified in COVID-19 autopsy tissues.

## Coronavirus Structure

**Figure 1:**
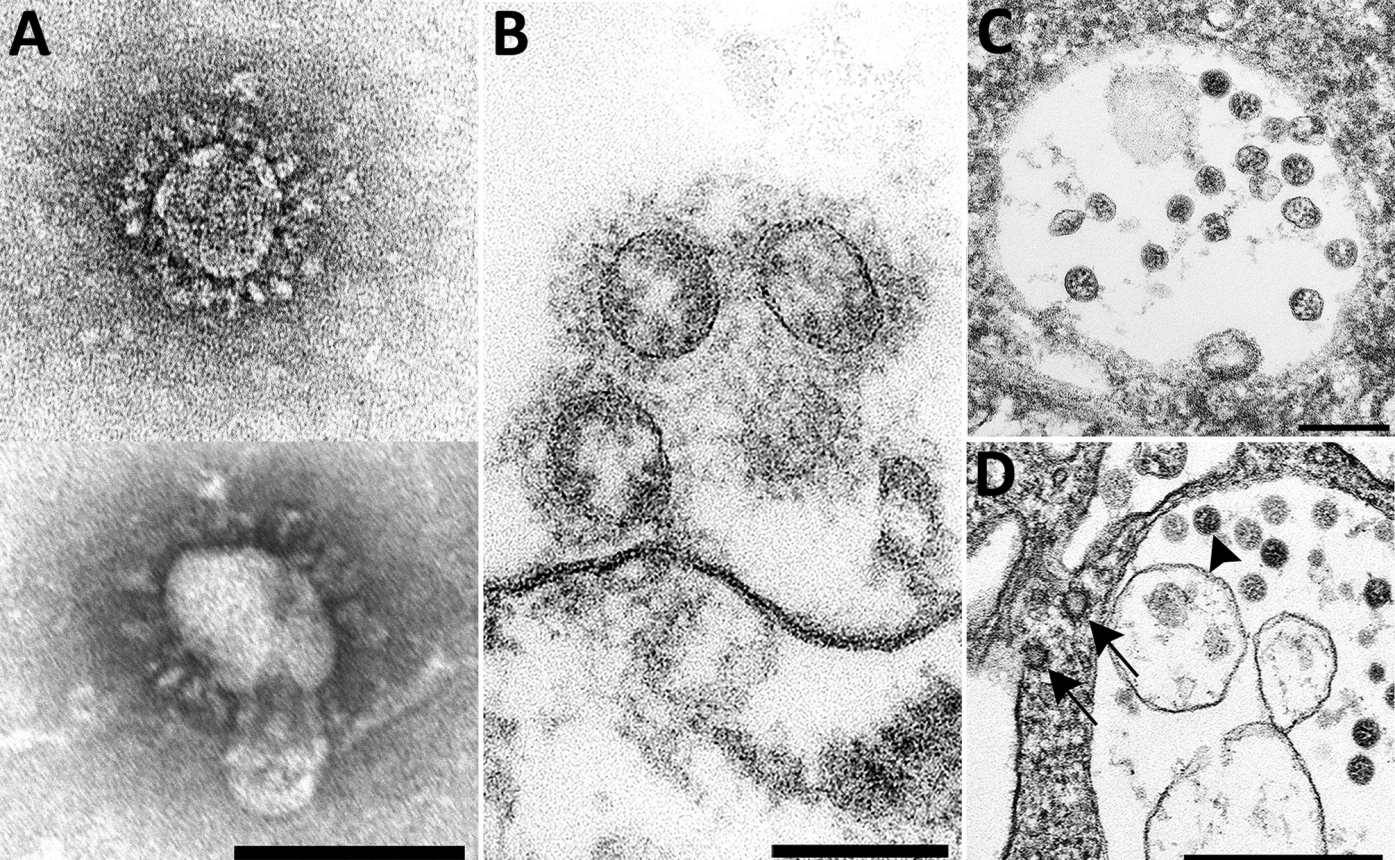
Overview of the ultrastructural features of coronavirus morphology as seen by negative stain and thin section. A) Extracellular viral particles ≈100 nm in diameter with prominent peplomers (spikes). Prepared from a cell culture sample by negative stain using heavy metal salt solutions to coat the outside of the virus. Scale bar indicates 100 nm. B) Extracellular viral particles ≈100 nm in diameter with clearly visible spikes. Cross sections through the helical nucleocapsid are visible on the interior of the particle as electron-dense black dots, 6–12 nm in diameter. Prepared by thin section from a formalin-fixed autopsy specimen. Scale bar indicates 100 nm. C) Intracellular viral particles ≈80 nm in diameter held within a membrane-bound vacuole. Cross sections through the helical nucleocapsid are visible inside the particles. Prepared by thin section from a formalin-fixed autopsy specimen. Scale bar indicates 200 nm. D) Intracellular viral particles (arrowhead) within a membrane-bound vacuole and nearby clathrin-coated vesicles (CCV) in the cytoplasm (arrows). CCV spikes directly contact the cell cytosol; viral spikes, barely visible as a faint fuzz, contact the vacuole contents. Cross sections through the helical nucleocapsid are visible inside the viral particles but not within the CCVs. Prepared by thin section from a glutaraldehyde-fixed cell culture sample. Scale bar indicates 500 nm.

Knowledge of coronavirus ultrastructure and morphogenesis is paramount to avoiding errors in identification. The name coronavirus was coined by June D. Almeida, who visualized the virus by EM in 1967 (*35*). The name was derived from the surface peplomers or spikes that give the viral particles the appearance of having a solar corona. These spikes are one of the more distinctive features for a coronavirus. For diagnostic EM, coronaviruses can be observed using 2 techniques, negative stain (*36*) and thin section (*36**,**37*). Negatively stained samples are prepared by adsorbing virus suspended in fluid onto a plastic-coated grid, wicking off excess liquid, and staining with a heavy-metal salt solution. The virus is coated with the stain, which penetrates between spikes protruding on the virus surface, making them visible. Thus, negative stain EM images readily show the prominent spikes that are associated with coronaviruses (Figure 1, panel A). For thin section EM, tissues or infected cell culture specimens are fixed in formalin or glutaraldehyde, stained with osmium, embedded in epoxy resin, baked to harden, and sectioned using an ultramicrotome. The resulting ultrathin sections show a cross-sectional view of the cells and viruses. In ultrathin sections of fixed tissues, coronavirus particles are ≈100 nm in diameter including their peplomer spikes and ≈80 nm in diameter excluding spikes (Figure 1, panel B). The spikes on coronavirus particles within cytoplasmic vacuoles (Figure 1, panel C) are not easily visible by thin section EM, unless the tissue is processed with tannic acid; instead, they usually appear as a fuzz on the surface of the virus. The difference in the appearance of the virus in negative stain versus thin section contributes to the confusion and misidentification of coronaviruses. Spikes are very rarely as clear in thin-sectioned specimens as they are when seen by negative stain EM.

## Coronavirus Biology

Proper identification of coronaviruses within tissue samples requires understanding the biology of the virus and its replicative process (*37*–*39*); this knowledge ensures that the microscopist is searching for it in the correct cellular location, saving valuable time and helping to avoid misidentifying normal cellular structures as virus. In an infected cell, virus replication takes place within the host cell cytoplasm. Several studies have documented that the coronavirus replicative process induces formation of modified host cell membranes, including structures like double-membrane vesicles and convoluted membranes (*39*,*40*). Coronavirus structural components, including envelope, membrane, and spike proteins, are inserted into the endoplasmic reticulum (ER) and eventually move to the endoplasmic reticulum–Golgi intermediate compartment (ERGIC) (*37*,*41*). Complete virions are produced when the helical viral nucleocapsids bud through membranes of the ERGIC, taking with them ERGIC membrane, which pinches off to form spherical viral particles inside vesicles; the budding process provides the viral envelope (*37**,**38*). This region between the rough ER (RER) and the Golgi complex is known as the budding compartment. Virions then accumulate in the intracisternal space that forms a vacuole; if spikes were visible, they would be observed within the area of this membrane-bound vacuole (Figure 1, panels C, D). The vacuoles with viral particles migrate to the cell surface where the vacuolar and plasma membranes fuse, and the virus is extruded, resulting in extracellular particles in which spikes may be more apparent (*38*) (Figure 1, panel B). Of note, accumulations of coronavirus would not be found free within the cytoplasm of a cell, and at no point would the spikes of a coronavirus be in direct contact with the cytosol.

## Structures Commonly Misidentified as Coronaviruses

**Figure 2:**
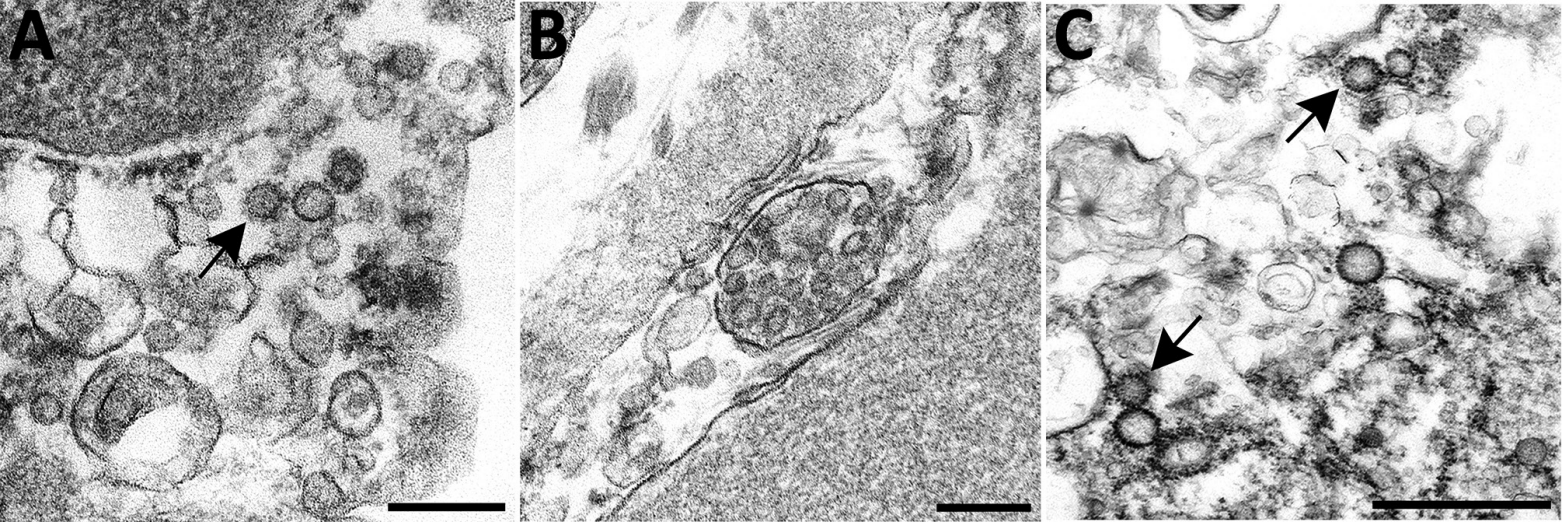
Overview of differential ultrastructural features of subcellular structures commonly misidentified as coronaviruses; all were prepared by thin section from formalin-fixed autopsy specimens. A) Clathrin-coated vesicles (CCVs), circular vesicles with a fringe of clathrin protein (arrow), in the cell cytoplasm range in size from 60 nm−100 nm. Differentiation: clathrin surrounding the vesicle may be misinterpreted as viral spikes, however, CCVs are free in the cell cytoplasm, and clathrin is in direct contact with the cytoplasm. Intracellular coronaviruses are found within membrane-bound vacuoles, and spikes, if visible, are in contact with the vacuolar contents. CCVs lack the internal black dots that signify cross sections through the viral nucleocapsid. Scale bar indicates 200 nm. B) Multivesicular body (MVB), a collection of membrane-bound roughly spherical vesicles formed by the inward budding of an endosomal membrane. Differentiation: MVBs may be confused with a vacuolar accumulation of coronavirus particles. Vesicles within multivesicular bodies do not have internal black dots that signify cross sections through the viral nucleocapsid. Scale bar indicates 200 nm. C) Circular cross sections through rough endoplasmic reticulum (RER) (arrows) found free within the cytoplasm. Differentiation: ribosomes along the endoplasmic reticulum may be confused with viral spikes. Ribosomes of vesiculating RER are in direct contact with the cell cytoplasm, unlike coronavirus spikes, which would be in contact with vacuolar contents. Vesiculating RER lacks cross sections through the viral nucleocapsid. Scale bar indicates 1 µm.

We performed a literature search for reports published during March 1–November 30, 2020, that used EM to identify coronavirus directly in patient specimens. We used the keywords ultrastructure or electron microscopy in conjunction with COVID-19, SARS-CoV-2, or coronavirus when searching Google Scholar, PubMed, MEDLINE, Web of Science, and Scopus. We identified 27 reports with EM findings. Four of these reports and 1 letter to the editor included correctly identified coronavirus (*30*–*34*). The other 23 articles revealed a pattern of subcellular structures misidentified as virus (Table), including clathrin-coated and coatomer-coated vesicles (CCVs; 48%), multivesicular bodies (MVBs; 26%), circular cross-sections through vesiculated RER (19%), spherical invaginations of RER (4%), and other nonviral structures (30%). Figure 2 shows an overview of these subcellular components observed within autopsy tissues.

The most common structures erroneously identified as coronaviruses were CCVs (Table; Figure 2, panel A), which play essential roles in cellular transport. The clathrin protein is associated with vesicle formation and transport at the plasma membrane and trans-Golgi network. Coatomer proteins mediate transport within the Golgi complex and between the Golgi complex and ER (*42*). Although the sizes of the CCVs and the virus may be similar, the cellular location of each and the lack of cross sections through the viral nucleocapsid are key differentiating features (Figure 1, panel D). CCVs are found free in the cytoplasm, not within the membrane-bound vacuoles where intracellular coronavirus particles are found (Figure 1, panel D). The clathrin or coatomer projections protruding from the vesicles as a fringe can be easily misinterpreted as viral spikes. These clathrin and coatomer proteins, however, are in direct contact with the cell cytosol (Figure 1, panel D; Figure 2, panel A), whereas spikes on intracellular coronavirus particles, if visible, are within the vacuolar contents and not the cell fluid (Figure 1, panels C, D). An additional morphologic feature visible in coronaviruses in thin section EM is the helical nucleocapsid (*41*,*43*), which can be seen in cross sections as electron-dense black dots 6–12 nm in diameter on the inside of the viral particles (Figure 1 panels B-D). CCVs do not contain these black dots (Figure 1, panel D; Figure 2, panel A). The lack of these dots in a subcellular structure is a good indicator that it is not coronavirus.

Several reports have misidentified multivesicular bodies (MVBs) as coronavirus particles (Table). MVBs are a type of late endosome consisting of multiple vesicles within a membrane-bound structure formed from the inward budding of an outer endosomal membrane (Figure 2, panel B) and are part of standard cellular processes for protein degradation (*23*,*24*). MVBs may be confused with vacuolar accumulations of coronavirus; both have the appearance of a membrane-bound collection of spherical particles (Figure 1, panels C, D; Figure 2, panel B). The key differentiating feature is the lack of cross sections through the viral nucleocapsid within the spherical profiles of the MVB. Any purported membrane-bound accumulation of virus-like particles without the black dots signifying cross sections through the viral nucleocapsid is likely an MVB rather than a vacuole containing coronavirus. MVBs have also been misidentified as double-membrane vesicles, a part of the replication complex for coronaviruses. However, double-membrane vesicles are composed of 2 tightly apposed membranes, which is not the case with MVBs (*37*,*40*). In a letter to the editor of the journal *Kidney International*, Calomeni et al. discussed the prevalence of MVBs in kidney biopsies from the pre–COVID-19 era (*24*).

Circular cross sections through vesiculated RER, with its ribosome-studded membranes, have also been highlighted as viral particles in tissue samples (Table). The RER is the site of protein synthesis and plays a role in viral replication; however, it has been misidentified as virus itself in some recent publications. A thin section through an area of RER may give the appearance of a circular membrane with small dark spikes along the outside edge of the membrane (Figure 2, panel C). In this instance, the spikes along the membrane are in fact ribosomes, not viral peplomers. The substantial variability in size of circular cross sections through the RER indicate that these are not viral particles; coronavirus particles with spikes are typically around 80–100 nm in diameter. Vesiculating RER also lacks the interior black dots of cross sections through the viral nucleocapsid, and the ribosomes, mistaken for spikes, are in direct contact with the host cell cytoplasm, rather than the vacuolar content.

An additional structure that has misled investigators appears to be an invagination of RER that results in roughly spherical particles with ribosomes inside (*13*), referred to in one paper as outside-in RER (*44*). These virus-like particles are uniform and comparable in size to coronaviruses, ≈100 nm in diameter. The particles meet the morphological criteria for a coronavirus except that the dots inside are larger (≈20 nm) than those in cross sections through coronavirus nucleocapsids (≈6–12 nm). The exact nature of their composition or relationship to any cellular processes has not been determined.

## Identifying Coronaviruses Using Formalin-Fixed Tissue and Formalin-Fixed, Paraffin-Embedded Samples

Although EM alone is a powerful tool, a multipronged approach for detecting and identifying viral particles can be key to the prompt and accurate diagnosis of the extent of infection and for further investigation into disease pathology. This fact is particularly true in rapidly developing situations, such as the COVID-19 pandemic, when transmission of high-quality scientific information is of paramount importance. Although formalin- or glutaraldehyde-fixed tissues embedded for EM are necessary for accurate classification of viral morphology and morphogenesis, the detection of virus within a tissue may require a more targeted approach to find the infected area, such as by working closely with a pathologist to select promising areas for EM from formalin-fixed tissues displaying evident disease pathology (e.g., areas of pulmonary consolidation) or by using formalin-fixed, paraffin-embedded (FFPE) blocks (*31*).

The benefit of using FFPE samples is the ability to perform a variety of diagnostic methodologies on serial sections from the same tissue, enabling comparison and correlation of test results. For example, immunohistochemistry (IHC) tests using antibodies for a specific pathogen can be applied to sections of FFPE tissue on glass slides and, based on the IHC results, areas of interest that are likely to contain the antigen or virus can be identified for EM analysis. The selected areas can be prepared for EM by embedding a tissue section 4–6 µm thick affixed to a glass side in situ on the slide (on-slide), or the targeted tissue can be removed from the FFPE block using a biopsy punch, deparaffinized, and embedded in epoxy (*31*,*36*). However, processing and analyzing each of these sample types presents challenges; the foremost is the accurate identification of viral particles, because the ultrastructural morphology of the virus and the surrounding tissue may be degraded by the processing for light microscopy. Having an area of interest selected that is already positive for a virus by another test, such as IHC or in situ hybridization (ISH), aids in viral detection and identification by EM. For this approach to be successful, IHC and ISH assays must be rigorously evaluated and validated by using negative controls and by testing antibody cross-reactivities to prevent false positives and the misinterpretation of nonspecific staining.

**Figure 3:**
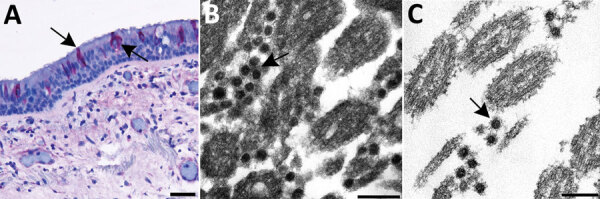
Use of immunohistochemistry and electron microscopy to detect severe acute respiratory syndrome coronavirus 2 (SARS-CoV-2) in formalin-fixed paraffin embedded (FFPE) autopsy tissues. A) Immunostaining (arrows) of SARS-CoV-2 in the epithelial cells of the trachea. Scale bar indicates 20 µm. B) Ultrastructural features of extracellular SARS-CoV-2 particles (arrow) in association with ciliated cells of the trachea from paraffin section in panel A, prepared using an FFPE on-slide method. Scale bar indicates 200 nm. C) Thin section of a biopsy punch from the original FFPE block in panel A showing viral particles (arrow) ≈75 nm. Scale bar indicates 200 nm.

An example of this approach in autopsy tissues is shown in Figure 3. We selected an area of interest for EM based on a positive IHC result for SARS-CoV-2 in ciliated epithelial cells from the trachea (Figure 3, panel A). Using FFPE samples leads to compromised ultrastructure and a reduction in viral particle size because of the additional processing these samples undergo, including embedding in paraffin, deparaffinizing, staining, and drying, before being dehydrated and embedded in epoxy for EM. This deteriorated ultrastructure is particularly evident in the on-slide sample (Figure 3, panel B). Although morphology is compromised, the presence of large numbers of intracellular and extracellular uniformly sized particles in areas corresponding to positive immunostaining or molecular labeling are clues to the presence of viruses. The extracellular viral particles are smaller than would be observed in a typical glutaraldehyde-fixed thin section sample due to shrinkage from processing, closer to 75 nm in diameter than 100 nm, but can still be differentiated from the surrounding ciliary structures. Closer examination of the FFPE biopsy punch sample (Figure 3, panel C) reveals apparent cross sections through the viral nucleocapsid as well as a surrounding fuzz which is suggestive of peplomers. Using a multifaceted approach such as this for SARS-CoV-2 detection enables accurate determination of the localization of the virus within tissues and correlation of histopathological with ultrastructural features of SARS-CoV-2 infection.

## Conclusions

EM is powerful in its ability to provide a window into the ultrastructure, and thus function, of tissues and the infectious agents they may contain. It gives scientists a valuable tool to provide clear visual evidence of viral infection and disease pathology, unlike biochemical tests that require choosing a priori the correct reagent and may yield false positive or false negative results. However, knowledge of both viral morphogenesis and normal subcellular architecture is necessary to identify viruses correctly by EM. The issue of virus misidentification within tissue samples is not unique to coronaviruses or limited to those subcellular structures we addressed in this report; nuclear pores may be mistaken for herpesvirus, perichromatin granules for smaller DNA viruses such as parvoviruses or polyomaviruses, and ribosomes for picornaviruses. Neurosecretory granules and glycocalyceal bodies can also be misidentified as viruses (*23*,*45*,*46*). Before declaring the presence of viruses, particularly complex enveloped viruses with multiple appearances in different stages of maturation, we recommend consulting with a trained diagnostic EM professional who has extensive knowledge of viral ultrastructure. If, after such consultation, a definitive identification still cannot be made, a descriptive report may be used, including the size, morphology, and cellular location of the particles of interest. One should only use the term virus or a more specific term, such as coronavirus, when the particles in question can be positively identified. The term virus-like may be used when only some morphologic criteria for virus identification have been met or in cases of deteriorated ultrastructure.

The use of diagnostic EM for infectious diseases pathology research is at its best when it involves collaboration between specialists in pathology, microscopy, and microbiology. The scientific community’s interest in diagnostic EM and the need for trained professionals in this field is highlighted by the number of recent articles seeking to identify SARS-CoV-2 particles in patient specimens. In each case of erroneously identified coronavirus particles, the structures mistaken for virus are common cellular organelles. These misinterpretations are easy to make without extensive training and are made easier by the publication of incorrectly identified viral structures. Articles with misidentified viral particles are used by others to verify the presence of viral particles in their own research, potentially wrongfully documenting the presence of the virus in damaged tissues. However, rather than actual virus infection of a failing organ, the damage could be due to lack of support of the infected organ due to the body’s response to toxins, such as cytokines and circulating debris, or to clotting (*47**,**48*). These continued misinterpretations could have meaningful impacts on future publications about coronavirus detection and research.

Coronaviruses have been the cause of 3 life-threatening human disease outbreaks over the past 18 years: SARS-CoV in 2002, Middle East respiratory syndrome coronavirus in 2012, and finally, the 2020 SARS-CoV-2 pandemic. Given the abundance of coronaviruses in the natural environment, the scientific literature should accurately reflect the nature of SARS-CoV-2 infection, including the ultrastructure and cellular location within the cell of the virus. This need is important not only for our current understanding of SARS-CoV-2 infection but also as the public health community prepares for future outbreaks. Diagnostic EM has played a key role in previous outbreaks of Nipah virus, SARS-CoV, monkeypox virus, and Ebola viruses, as well as many others (*49*), and will continue to be a tool for detecting and characterizing new and emerging pathogens.
